# Evaluation of the platelet-to-white-cell ratio (PWR) as predictor of long-term all-cause mortality in patients with acute myocardial infarction

**DOI:** 10.1038/s41598-026-56805-x

**Published:** 2026-06-09

**Authors:** Michael Kraus, Timo Schmitz, Philip Raake, Jakob Linseisen, Christa Meisinger

**Affiliations:** 1 Epidemiology, Medical Faculty, University of Augsburg, Stenglinstraße 2, 86156 Augsburg, Germany; 2 Department of Cardiology, Respiratory Medicine and Intensive Care, University Hospital Augsburg, Augsburg, Germany

**Keywords:** Myocardial infarction, Mortality, Outcome, Platelet-to-white-cell-ratio, Platelets, Leukocytes, Cardiology, Epidemiology

## Abstract

The platelet-to-white-cell ratio has recently gained attention as a potential prognostic indicator. This study investigated the association between platelet-to-white-cell ratio at admission and long-term mortality in acute myocardial infarction patients, in comparison to leukocyte and platelet counts alone. The study included 4,964 patients aged 25–84 years with incident acute myocardial infarction hospitalized between 2010 and 2017, as recorded in the population-based Augsburg Myocardial Infarction Register. Platelet-to-white-cell ratio was calculated from admission platelets divided by leukocyte counts. Cox proportional hazards regression models were used to assess the associations between quartiles of platelet-to-white-cell ratio, of leukocytes, and of platelets with long-term all-cause mortality. During a median follow-up time of 4.7 years, 1,224 patients died. Platelet count in the highest quartile was observed to be associated with increased mortality risk (HR 1.32 (1.06–1.63). Platelet-to-white-cell ratio values were also found to be associated with risk of mortality. Multivariable adjusted HRs for the second, third, and fourth quartiles of platelet-to-white-cell ratio (vs. lowest quartile) were 0.64 (95% CI: 0.52–0.80), 0.83 (0.67–1.03), and 0.78 (0.62–0.98), respectively. Platelet-to-white-cell ratio at admission was associated with long-term mortality in patients with acute myocardial infarction. As a simple, cost-effective biomarker, platelet-to-white-cell ratio may support individualized risk stratification and clinical decision-making in acute myocardial infarction care.

## Introduction

The prognosis of a myocardial infarction depends on known predictors such as the time between the onset of symptoms and recanalization treatment, type of therapy, extent and localization of the myocardial damage and complications such as cardiac arrhythmia, heart failure and cardiogenic shock^1^. Furthermore, therapy adherence and secondary prevention play a central role^2^. In addition, it is known that inflammatory processes have an influence on the prognosis of myocardial infarction patients^3^. Increasingly, the interplay between platelet activity and inflammatory processes, which plays a role in both the development of atherosclerosis and the acute phase of myocardial infarction, is highlighted in this context^4^. Platelets and leukocytes are not only involved in plaque formation but also influence the course of myocardial infarction. Platelets are crucial for the formation of the initial thrombus, while leukocytes increase the tissue damage caused by oxygen deficiency through the release of pro-inflammatory cytokines^5,6^. Abnormal platelet or leukocyte counts are associated with a less favorable prognosis after acute myocardial infarction (AMI)^7,8^. An increase in platelet counts may increase the risk of stent thrombosis^9^, while decreased platelet counts may be a risk factor for reinfarction^10^. Similarly, elevated white blood cell counts are often associated with a systemic inflammatory response and are related to higher mortality^11^. Against this background, the platelet-to-white-cell-ratio (PWR) is more and more being investigated as a possible marker for clinical outcome of various diseases, such as cardiovascular and infectious diseases^12^. Due to its ability to reflect both thrombotic and inflammatory processes, it could be also a valuable predictive parameter for prognosis assessment and clinical decision-making in AMI patients. Therefore, the purpose of the present study was to investigate the prognostic relevance of PWR in comparison to leukocytes and platelets regarding long-term mortality in AMI patients using data from the population-based Augsburg Myocardial Infarction RegisterR.

## Methods

Data from the Augsburg Myocardial Infarction Register was used for this study. The register has existed since 1984 and was set up as part of the MONICA project (Monitoring Trends and Determinants in Cardiovascular Disease). Between 1996 and 2020, it continued as KORA Myocardial Infarction Register. In 2021, the project was fully transferred to the Augsburg University Hospital. The study area includes the city of Augsburg and the two neighboring districts with a total population of around 705,000 people. All cases of hospitalized AMI were recorded in the register if the patients survived longer than 24 hours after admission, were between 25 and 84 years old (since 2009) and had their main residence in the study area. Detailed information on participant recruitment and data collection can be found in previous publications^13^.

All acute incident AMI cases that were treated in one of the participating hospitals between 2010 and 2017 and recorded by the register were included in the current analysis. Of a total of 6,325 patients, 926 were excluded due to missing information on relevant covariables, leaving 6,137 patients. Patients who died within the first 28 days after the event (*n* = 435) were also excluded to focus purely on long-term mortality. Finally, 4,964 patients aged 25 to 84 years with an incident hospitalized AMI could be included in the analysis.

The study was approved by the Ethics Committee of the Bavarian Medical Association and complies with the requirements of the “Declaration of Helsinki”^14^. A written informed consent was obtained from all subjects. The study was registered at the German Register of Clinical Studies (DRKS, project number DRKS00029042).

### Data collection

During the hospital stay, trained nurses conducted personal interviews with the patients using a standardized questionnaire. In addition, data were collected from the patients’ medical records, such as information on sociodemographic characteristics, risk factors, comorbidities, diagnostic procedures and invasive and non-invasive treatments.

The admission ECG was analyzed by a physician, and each case was assigned to one of the following three groups: ST-elevation myocardial infarction (STEMI), non-ST-elevation myocardial infarction (NSTEMI), bundle branch block ECG. Estimated glomerular filtration rate (eGFR) was calculated using the admission creatinine values according to the CKD-EPI formula^15^. The eGFR was divided into the following categories: ‘normal renal function’ (eGFR ≥ 60 mL/min/1.73 m2), ‘mildly impaired renal function’ (eGFR between 30 and 59 mL/min/1.73 m2), ‘severely impaired renal function’ (eGFR < 30 mL/min/1.73 m2). For the left ventricular ejection fraction (EF), the categories “severely impaired left ventricular EF” (≤ 30%) and “not severely impaired EF” (> 30%) were built. One categorical variable for the invasive treatment was created including three levels: percutaneous coronary intervention (PCI), coronary artery bypass graft (CABG), and no invasive therapy. If a patient underwent both PCI and CABG, he/she was included in the CABG group, as this represented the definitive treatment. Finally, medications at hospital discharge were documented, in particular: antiplatelets, ACE inhibitor or angiotensin-2 receptor blocker (ARB), betablockers and statins.

### Exposures

At admission due to an AMI, usually within 15 min, venous blood samples were obtained from the patients for measurement of peripheral blood cell analysis. In the present analysis the admission blood parameters leukocyte count (/nL) and admission platelet count (/nL) were considered. PWR was calculated by dividing the amounts of platelets (x10^9^/L) divided by leukocyte values (x10^9^/L).

### Outcome

The outcome of this study was long-term all-cause mortality. Mortality was ascertained by regularly checking the vital status of all registered AMI patients in cooperation with the regional population registries. Death certificates were obtained from local health departments. The last complete mortality follow-up was performed in 2019.

### Statistical analysis

Categorical variables are represented by totals and percentages, and the chi-square test was used to test for differences. Continuous variables are represented by median and IQR and the Mann-Whitney U test was used to test for group differences. The assumption of normal distribution was verified using Shapiro-Wilk test and visually by inspecting histograms and Q-Q plots. The study population was stratified into quartiles of the exposure variables leukocytes, platelets, and PWR. The survival time of the different groups were visualized graphically using Kaplan-Meier survival curves. A log-rank test was conducted to test for differences between survival curves.

### Cox regression

Relative risks of all-cause long-term mortality were computed for quartiles 2, 3, and 4 as compared with the lowest quartile (reference group) in separate Cox proportional hazards models, one for each exposure measure. Confounders were identified based on literature research; the final models were adjusted for sex (categorical), age (continuous), chest pain status (categorical), admission ECG pattern (categorical), diabetes (categorical), smoking status (categorical), eGFR (continuous), invasive therapy (categorical), peak creatine kinase (CK) MB value, therapy with antiplatelets (categorical), ACE inhibitor or ARBs (categorical), betablockers (categorical) and statins (categorical) at discharge. The continuous variables age, BMI, and peak CKMB values were tested for linearity in the Cox regression by including a squared term. For age and BMI, the squared terms were significant and therefore also included in the final models. Furthermore, a formal test for interaction with sex was conducted, yet no interaction was found. Cox regressions with time-dependent covariables were used to test the proportional hazards assumption for each variable. No violation of proportional hazards was observed.

The analyses were conducted using IBM SPSS (version 29.0).

## Results

Altogether 4,964 patients with an incident AMI (3,534 men, 1,430 women) could be included in the analysis. During a median follow-up period of 1,702 (IQR: 988; 2,477) days 1,224 patients died.

In Table 1 the characteristics of the study sample stratified by PWR quartiles are shown. Patients in higher PWR quartiles tended to be older, had lower admission leukocyte values, lower maximum CKMB values, and higher admission platelet counts. The proportion of females increased with increasing quartiles of PWR. Patients with higher PWR values were less likely to have hypertension and were less frequently current smokers; they less often suffered from diabetes and reduced LVEF. Furthermore, patients with higher PWR were more frequently presented with typical chest pain, hyperlipidemia, and an NSTEMI infarction. A higher percentage of patients in quartile 4 of PWR compared to lower quartiles received beta-blockers and ACE-inhibitors/ARBs at discharge.

**Table 1:** Characteristics of the study sample by quartiles of platelet-to-white-cell-ratio (PWR). Categorical variables are given as total numbers and %, continuous variables as median (IQR). N represents the number of patients without missing values.

Characteristics	PWR					
	*N*	Quartile 1	Quartile 2	Quartile 3	Quartile 4	*p*-value
		1–≤17	17–<23	23–<29	29–195	
**Died at follow-up**	4,875					< 0.001
Yes		397 (32.6)	260 (21.3)	272 (22.3)	273 (22.4)	
No		821 (67.4)	959 (78.7)	947 (77.7)	946 (77.6)	
**Sex**	4,875					< 0.001
Males		958 (78.7)	901 (73.9)	842 (69.1)	773 (63.4)	
Females		260 (21.3)	318 (26.1)	377 (30.9)	446 (36.6)	
**Age (years)**	4,964	67 (56;76)	67 (56;75)	68 (58;76)	69 (59;76)	< 0.001
**BMI (kg/m²)**	4,837	27.0 (24.4;30.1)	27.1 (24.4;30.3)	27.1 (24.4;30.5)	26.8 (24.2;29.8)	0.429
**Prehospital time (minutes)**	4,487	156.5 (82;455.75)	155 (81;510)	155 (84;528.5)	182 (86;713)	0.055
**Laboratory parameters**						
Admission leukocytes (/nL)	4,885	13.9 (11.3;16.8)	10.9 (9.2;12.8)	9.2 (7.8;10.9)	7.5 (6.3;9.0)	< 0.001
Admission platelets (/nL)	4,882	226.0 (189.0;272.0)	217.0 (186.0;255.0)	234.0 (199.0;275.0)	268.0 (225.0;318.0)	< 0.001
Platelet-to-white-cell-ratio	4,880	14.4 (12.3;15.9)	20.0 (18.9;21.3)	25.4 (24.0;26.9)	34.7 (31.3;40.0)	< 0.001
Highest CKMB values (U/l)	4,952	106 (48;244.5)	82 (40;190.75)	55 (31;129)	42 (25;91)	< 0.001
eGFR (CKD-EPI)	4,841					< 0.001
eGFR ≥ 60 ml/min/1.73 m²		760 (62.7)	843 (69.4)	876 (72.5)	893 (74.1)	
eGFR 30–59 ml/min/1.73 m²		359 (29.6)	317 (26.1)	287 (23.8)	269 (22.3)	
eGFR < 30 ml/min/1.73 m²		94 (7.7)	55 (4.5)	45 (3.7)	43 (3.6)	
**Hypertension**	4,875					0.009
Yes		901 (74.0)	939 (77.0)	969 (79.5)	954 (78.3)	
No		317 (26.0)	280 (23.0)	250 (20.5)	265 (21.7)	
**Diabetes**	4,875					0.002
Yes		417 (34.2)	368 (30.2)	388 (31.8)	332 (27.2)	
No		801 (65.8)	851 (69.8)	831 (68.2)	887 (72.8)	
**Smoking status**	4,680					< 0.001
Current smoker		491 (42.8)	435 (36.9)	331 (28.0)	256 (21.9)	
Ex-Smoker		331 (28.8)	369 (31.3)	383 (32.4)	427 (36.5)	
Never-Smoker		326 (28.4)	376 (31.9)	467 (39.5)	488 (41.7)	
**Hyperlipidemia**	4,875					< 0.001
Yes		594 (48.8)	679 (55.7)	700 (57.4)	704 (57.8)	
No		624 (51.2)	540 (44.3)	519 (42.6)	515 (42.2)	
**In hospital therapy**						0.022
PTCA		885 (72.8)	925 (75.9)	918 (75.4)	879 (72.2)	
Bypass		152 (12.5)	163 (13.4)	161 (13.2)	161 (13.2)	
No invasive therapy		179 (14.7)	131 (10.7)	138 (11.3)	178 (14.6)	
**Chestpain at admission**	4,850					< 0.001
Yes		888 (73.9)	1035 (85.2)	1017 (83.6)	1023 (84.1)	
No		314 (26.1)	180 (14.8)	200 (16.4)	193 (15.9)	
**Admission ECG pattern**	4,736					< 0.001
STEMI		592 (50.1)	533 (44.8)	395 (33.2)	312 (26.5)	
NSTEMI		507 (42.9)	593 (49.9)	708 (59.6)	785 (66.7)	
Bundle branch block		83 (7.0)	63 (5.3)	85 (7.2)	80 (6.8)	
**LVEF**	4,493					< 0.001
≤ 30%		108 (9.6)	73 (6.5)	57 (5.0)	45 (4.1)	
> 30%		1012 (90.4)	1057 (93.5)	1091 (95.0)	1050 (95.9)	
**Antiplatelets at discharge**	4,654					0.625
Yes		1150 (98.0)	1150 (98.6)	1147 (98.5)	1130 (98.3)	
No		24 (2.0)	16 (1.4)	18 (1.5)	19 (1.7)	
**ACE or ARBs** **at** **discharge**	4,652					0.012
Yes		1016 (86.5)	1028 (88.2)	1031 (88.6)	969 (84.4)	
No		158 (13.5)	138 (11.8)	133 (11.4)	179 (15.6)	
**Betablocker at discharge**	4,654					0.009
Yes		1104 (94.0)	1100 (94.3)	1111 (95 − 4)	1058 (92.1)	
No		70 (6.0)	66 (5.7)	54 (4.6)	91 (7.9)	
**Statins at discharge**	4,654					0.077
Yes		1070 (91.1)	1087 (93.2)	1093 (93.8)	1065 (92.7)	
No		104 (8.9)	79 (6.8)	72 (6.2)	84 (7.3)	

### Kaplan-Meier survival analyses

**Fig. 1:**
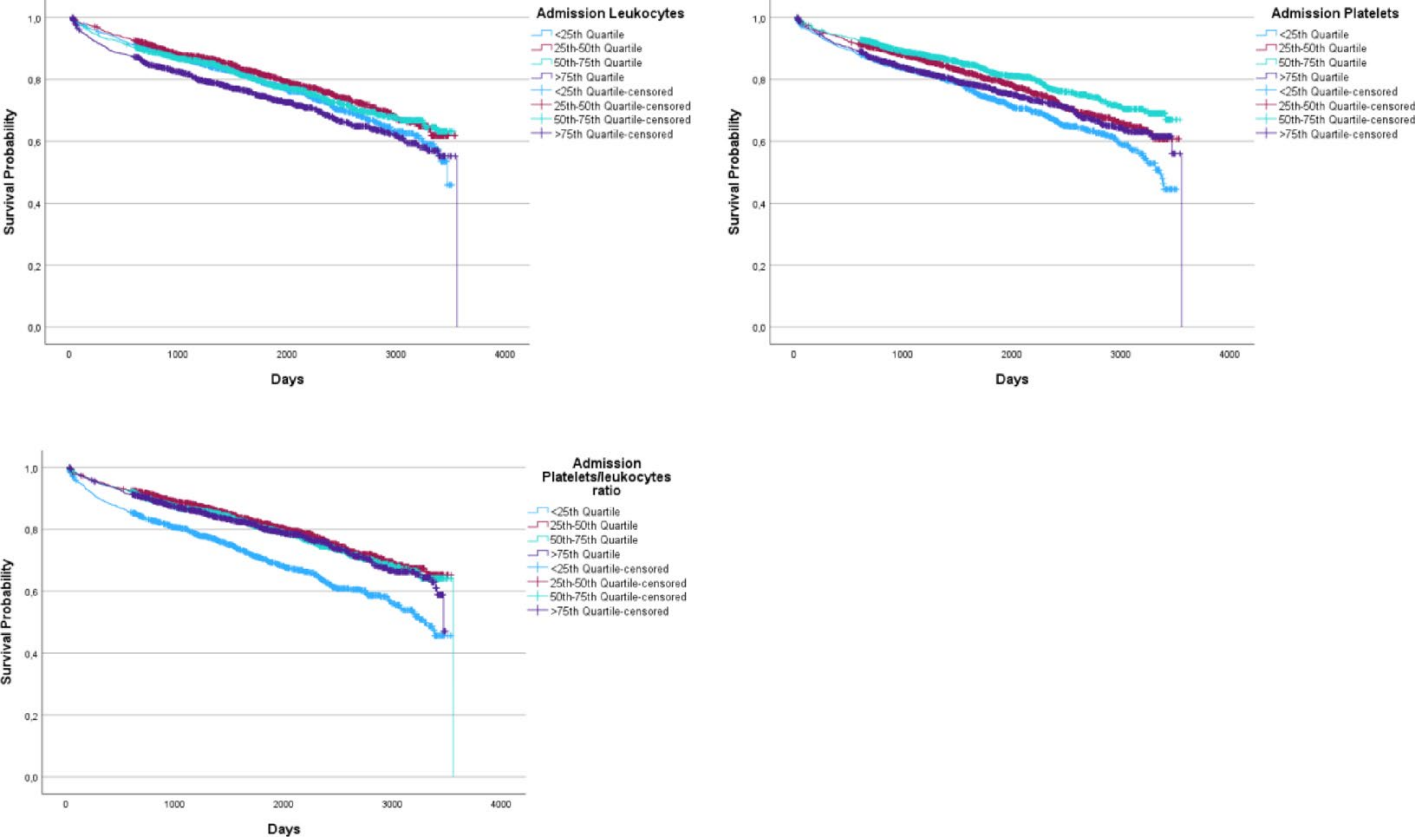
Kaplan-Meier survival curves by admission leukocytes, platelets, and platelets/leukocytes ratio quartiles, patients aged 25–84 years. Log-rank test: *p*<.001 for all three blood values.

Figure 1 shows the unadjusted Kaplan-Meier survival curves for all three exposures stratified by quartiles. Here too, patients with leukocyte and platelet values in the top quartile had a lower probability of survival than patients in the bottom quartile. In contrast, for patients with PWR values in the first quartile the lowest survival probability over time was observed.

### Results of the Cox regression analysis

Admission leukocyte values were not associated with a significantly elevated mortality risk (Table 2.). However, patients with admission platelet count in the highest quartile had a significantly increased risk of all-cause mortality compared to those in the lowest quartile (HR 1.32; 95% CI 1.06–1.63, p value: 0.011), while no significant increase in mortality risk was observed in patients for the second and third quartiles.

**Table 2:** Results from the Cox-proportional regression models analyzing the association between the three exposures (leukocytes, platelets, and platelet-to-white-cell ratio) and all-cause long-term mortality.

	Deaths/N	HR (95% CI)	p-value
Leukocytes			
Q1 (≤7.9/nL)	302/1240	1.0	
Q2 (8–10/nL)	274/1234	0.90 (0.71–1.14)	0.379
Q3 (10.1–12.8/nL)	297/1253	1.16 (0.93–1.46)	0.192
Q4 (≥12.9/nL)	351/1237	1.23 (0.97–1.56)	0.090
Platelets			
Q1 (≤186/nL)	353/1164	1.0	
Q2 (187–224/nL)	285/1238	0.97 (0.78–1.20)	0.779
Q3 (225–271/nL)	246/1260	0.87 (0.70–1.09)	0.239
Q4 (≥272/nL)	340/1302	1.32 (1.06–1.63)	0.011
Platelet-to-white-cell ratio			
Q1 (≤17)	397/1218	1.0	
Q2 (17–<23)	260/1219	0.64 (0.52–0.80)	<0.001
Q3 (23–<29)	272/1219	0.83 (0.67–1.03)	0.088
Q4 (≥29)	273/1219	0.78 (0.62–0.98)	0.035

Regarding PWR, all-cause mortality risk was significantly lower in the second (HR 0.64; 95% CI 0.52–0.80, p value: <0.001) and fourth (HR 0.78; 95% CI 0.62–0.98, p value: 0.035) quartiles compared to the first quartile, with the third quartile showing a non-significant trend toward lower risk (HR 0.83; 95% CI 0.67–1.03, p value: 0.088) after multivariable adjustment (Table 2.).

## Discussion

In the present study, we found that the PWR at admission was associated with long-term mortality in patients with AMI. Patients in the first quartile of PWR had the highest mortality, while those in the second (HR 0.64) and fourth (HR 0.78) quartiles had a significantly reduced risk. The third quartile showed a non-significant trend towards lower risk (HR 0.83) compared to the first quartile. In contrast, leukocyte and platelet counts alone were less predictive, with a significant increase in mortality observed only among patients with platelet counts in the highest quartile.

A low PWR may indicate heightened systemic inflammation coupled with a limited thrombotic reserve, such as thrombocytopenia and/or leukocytosis. Previous studies have shown that extreme platelet levels on admission are associated with increased long-term mortality^7,16^, and leukocytosis is linked to poor prognosis after AMI^8^. In a prior study on STEMI patients, leukocytosis after primary PCI was associated with greater myocardial edema, lower myocardial recovery, larger infarct size, and worse medium-term outcomes^17^. White blood cell count has also been identified as an independent predictor of no-reflow and in-hospital mortality in 1,016 Japanese patients undergoing PCI^18^. Furthermore, Song et al.^19^ found, that admission platelet count has a U-shaped association with all-cause mortality two years after AMI. Very low platelet counts may reflect consumptive coagulopathy or severe illness, while very high counts may indicate heightened thrombotic potential. In our study, by combining platelet and leukocyte counts, PWR may better reflect the balance between thrombotic potential and inflammatory activation, mitigating some limitations of individual markers. Nonetheless, extreme platelet values could have disproportionately influenced PWR in the present investigation.

To our knowledge, this is the first analysis investigating the association between PWR and long-term mortality in AMI patients. Prior studies suggested a protective role of higher PWR in cardiovascular conditions. For example, STEMI patients with a PWR of 24.4 ± 8.9 (vs. 21.6 ± 7.6) showed a higher rate of spontaneous reperfusion, lower in-hospital mortality, and fewer complications^20^. Another study observed, that a PWR < 15.88 in acute decompensated heart failure patients was associated with a significantly increased 30-day mortality^21^. These findings support the concept of PWR as an integrative biomarker reflecting both inflammatory and thrombotic processes.

The differences in STEMI prevalence and severely reduced LVEF across PWR quartiles observed in the present study suggest that PWR is associated with AMI severity. Larger infarcts trigger stronger inflammatory responses and more pronounced hematological changes, including leukocytosis and platelet alterations^22,23^. Thus, PWR may partly reflect the extent of myocardial injury and acute disease severity, while also providing prognostic information^21^. Individual variability in inflammatory and thrombotic susceptibility may further influence both early outcomes and long-term cardiac remodeling^23,24^. Further studies are necessary to clarify the relative contribution of myocardial injury and systemic host response in this connection.

In the present study, the Kaplan–Meier curves diverged immediately after the acute event, suggesting that PWR mainly reflects early post-infarction pathophysiology, including heightened inflammatory activation and thrombotic burden^20^. However, the curves remained separated throughout follow-up, suggesting that PWR not only captures acute disease severity but may also reflect biological mechanisms relevant to long-term outcomes, such as ventricular remodeling and chronic cardiovascular risk.

Although no effect modification by sex was observed in this study, differences in PWR between males and females may reflect underlying biological variation in platelet and leukocyte counts. Women generally have higher platelet counts than men, particularly before menopause, likely due to hormonal influences on hematopoiesis and immune function, as well as factors such as menstrual blood loss leading to reactive thrombopoiesis^25,26^. Differences in total white blood cell counts between men and women are generally smaller and less consistent, though distributions of specific leukocyte subsets may vary with age and hormonal status^27,28^. Additionally, hematologic parameters are influenced by genetic and environmental factors, including cytokine activity, thrombopoietin signaling, and ancestry^25^.Mechanistically, PWR reflects both quantitative and qualitative interactions between platelets and leukocytes, two central determinants of infarct size and complications^29^. Interestingly, the PWR was found to have the strongest association with all-cause mortality in cardiovascular patients compared to other hematological parameters such as the NLR (neutrophil-to-lymphocyte ratio) and PLR (platelet-to-lymphocyte ratio)^12^. A low PWR, reflecting disproportionately elevated leukocyte counts relative to platelets, may indicate heightened systemic inflammation and adverse prognosis^30,31^. Conversely, higher PWR values may reflect a more balanced inflammatory–thrombotic response^32^. Given the role of leukocytes in post-infarction healing and ventricular remodeling^33^, PWR may also correlate with long-term structural cardiac changes and thereby influence long-term mortality. In summary, these characteristics make PWR a low-cost and readily available parameter that may be useful for risk stratification in AMI patients. Integration into established risk scores could also be considered in future studies. Future studies should investigate whether and to what extent PWR can serve as a decision-making aid for further measures - for example regarding the need for intensive medical monitoring or additional measures for secondary prevention.

### Strengths and limitations

The strengths of this population-based study include complete enrollment, a large number of AMI cases, and standardized data assessment. The substantial sample size provided sufficient statistical power. A variety of different information and data was collected for each patient that allowed to adjust for various confounder and covariables in Cox regression analyses.

The present study has also several limitations. First, it is possible that we did not include all confounders affecting the association between the exposure variables and long-term mortality; unmeasured or residual confounding cannot be entirely excluded. In particular, no data on hereditary history or hormone levels was available. Also, no information regarding leukemia that occurred among the included patients was collected in this study. LVEF was measured during the hospital stay, but not at strictly predetermined and comparable times (e.g. shortly before discharge from hospital) for the different patients. Only patients aged 25 to 85 years were included in this study. Furthermore, no information on ethnicity was available. Therefore, the results might not be generalizable to all ethnicities and age groups.

## Conclusion

As a simple, cost-effective marker, PWR may support early risk stratification by identifying patients with high long-term mortality risk and be useful in clinical decision-making by complementing traditional prognostic indicators in AMI care. Future studies should explore its utility as a dynamic marker during the clinical course and its integration into personalized therapeutic strategies.

## Data Availability

The datasets generated during and/or analysed during the current study are not publicly available but are available in an anonymized form from the corresponding author on reasonable request.
